# ZEB1 and IL-6/11-STAT3 signalling cooperate to define invasive potential of pancreatic cancer cells via differential regulation of the expression of S100 proteins

**DOI:** 10.1038/s41416-019-0483-9

**Published:** 2019-05-24

**Authors:** Qais Al-Ismaeel, Christopher P. Neal, Hanaa Al-Mahmoodi, Zamzam Almutairi, Ibtihal Al-Shamarti, Kees Straatman, Nabil Jaunbocus, Andrew Irvine, Eyad Issa, Catherine Moreman, Ashley R. Dennison, A. Emre Sayan, Jonathan McDearmid, Peter Greaves, Eugene Tulchinsky, Marina Kriajevska

**Affiliations:** 1 Leicester Cancer Research Centre, University of Leicester, Leicester, UK; 2 University Hospitals of Leicester NHS Trust Hepato-Pancreato-Biliary Unit, Leicester, UK; 3 Centre for Core Biotechnology Services, University of Leicester, Leicester, UK; 4 Department of Cellular Pathology, Leicester Royal Infirmary, Leicester, UK; 5 Cancer Sciences Division, University of Southampton, Southampton, UK; 6 Department of Neuroscience, Psychology and Behaviour, University of Leicester, Leicester, UK; 7 Moscow Institute of Physics and Technology, Dolgoprudny, Moscow region Russia; 8 Department of Biomedical Sciences, Nazarbayev University School of Medicine, Astana, Kazakhstan; 9 Present Address: College of Medicine, University of Duhokl, Kurdistan region, Duhok, Iraq

**Keywords:** Pancreatic cancer, Cell invasion

## Abstract

**Background:**

S100 proteins have been implicated in various aspects of cancer, including epithelial-mesenchymal transitions (EMT), invasion and metastasis, and also in inflammatory disorders. Here we examined the impact of individual members of this family on the invasion of pancreatic ductal adenocarcinoma (PDAC) cells, and their regulation by EMT and inflammation.

**Methods:**

Invasion of PDAC cells was analysed in zebrafish embryo xenografts and in transwell invasion assays. Expression and regulation of S100 proteins was studied in vitro by immunoblotting, quantitative PCR and immunofluorescence, and in pancreatic lesions by immunohistochemistry.

**Results:**

Whereas the expression of most S100 proteins is characteristic for epithelial PDAC cell lines, S100A4 and S100A6 are strongly expressed in mesenchymal cells and upregulated by ZEB1. S100A4/A6 and epithelial protein S100A14 respectively promote and represses cell invasion. IL-6/11-STAT3 pathway stimulates expression of most S100 proteins. ZEB1 synergises with IL-6/11-STAT3 to upregulate S100A4/A6, but nullifies the effect of inflammation on S100A14 expression.

**Conclusion:**

EMT/ZEB1 and IL-6/11-STAT3 signalling act independently and congregate to establish the expression pattern of S100 proteins, which drives invasion. Although ZEB1 regulates expression of S100 family members, these effects are masked by IL-6/11-STAT3 signalling, and S100 proteins cannot be considered as bona fide EMT markers in PDAC.

## Background

Pancreatic ductal adenocarcinoma (PDAC) is one of the deadliest types of human cancer. High mortality is caused by late diagnosis, therapy resistance, immunosuppression, and high invasive potential, which often makes these tumours surgically incurable.^1^ Chronic pancreatitis (CP) represents a progressive disease of the pancreas with a strong fibrotic component. CP is characterised by persistent low-grade inflammation and increases the risk of PDAC 10-fold, indicating an association between inflammatory processes and the aetiology of PDAC. The key drivers of PDAC are activating mutations in *KRAS*, detected with 80–100% frequency in cancerous lesions.^1^ The vast majority of PDAC arise from pancreatic intraepithelial neoplasia (PanIN), and inflammation is an important mediator of the progression of pancreatic tumours. Implication of the epithelial-mesenchymal transition (EMT) in KRAS-driven PDAC development has been intensively studied in recent years.^2^

EMT and a reverse process, mesenchymal-epithelial transition (MET), are genetic programs important in normal embryonic development, and in tissue response to an injury.^3^ During EMT cells lose epithelial polarity, experience massive reorganisation of the cytoskeleton, acquire mesenchymal traits, and become motile and invasive. EMT/MET programs are determinants of cellular plasticity, they are reactivated in metastatic cancers facilitating tumour spread.^4^

EMT/MET programs are regulated by a number of signalling pathways, e.g., TGFβ or WNT, and also by inflammatory stimuli, such as IL-6 in colorectal cancer^5^ and IL-6/IL-8 in breast cancer.^6^ Several transcription factors, such as those belonging to the ZEB, SNAIL and TWIST families, execute EMT programs in normal and pathological conditions. The relevance of these factors to metastasis has been addressed in recent studies performed in the Pdx1-Cre/Kras^G12D^/P53^R172^^H/+^ mouse model of PDAC. Whereas deletion of *Snai1* or *Twist1* genes was dispensable for PDAC dissemination,^7^ knockout of *Zeb1* strongly reduced invasion and metastases in this mouse strain.^8^ Particular importance of ZEB1 for PDAC dissemination is in line with the previous observation that its presence in primary tumours significantly correlates with shortened overall patient survival.^9^

In vivo lineage tracing experiments have shown that a small proportion of Zeb1-positive invasive cells are detectable at early stages of pancreatic tumorigenesis in PanIN-bearing mice. These cells formed a pool of circulating tumour cells (CTCs) which possessed enhanced tumour-initiating potential and an ability to seed in the liver.^10^ Remarkably, formation of this cell population within PanIN and in the circulation could be blocked by the immunosuppressive agent dexamethasone, again indicating the importance of inflammatory signalling in PDAC. Circulating Zeb1-positive cells were characterised by enhanced expression of S100A4 (or Fsp1), a member of the S100 protein family implicated in EMT.^10^

The S100 family comprises 23 small calcium-binding proteins, most of which exert intra- and extracellular functions. In the human genome, 17 of the S100-encoding genes are located within a gene cluster at chromosome 1q21.3, referred to as the epidermal differentiation complex (EDC).^11^ S100 proteins have been implicated in various pathological conditions including cancer, cardiovascular diseases, fibrosis, and chronic inflammation. When released into the extracellular milieu by tumour cells, S100 proteins take part in the formation of the tumour microenvironment by attracting inflammatory cells.^12^ Inside cells, S100 proteins interact with their targets and affect various biological processes. Their most frequently reported role is in the control of cell migration and invasion via direct interaction with cytoskeletal components.^13,14^ One of the S100 family members, S100A4 is considered as a biomarker of EMT in several cancer types including PDAC^10,15^ and has been proven to play a role in cancer metastasis.^16^ The association between EMT and other members of the S100 protein family in pancreatic cancer remains less clear.

Here, we analysed the expression of S100 proteins in vitro and in PDAC samples and report that two family members only, S100A4 and S100A6, are associated with EMT and drive invasion of PDAC cells in vitro and in zebrafish embryo xenografts. In contrast, other members exhibited a more epithelial expression pattern, with S100A14 demonstrating a strong correlation with the epithelial phenotype in cell lines and in human PDAC samples. Accordingly, S100A14 repressed cell invasion and was required for the maintenance of the epithelial phenotype. Expression of S100 proteins is independently regulated by two signalling mechanisms, EMT/ZEB1 and IL-6/11-STAT3. While IL-6/11-STAT3 enhances the expression of most S100 proteins, ZEB1 activates S100A4/A6, but decreases expression levels of other family members including S100A14. ZEB1 synergises with IL-6/11-STAT3 in activating S100A4/A6, but counteracts the effect of inflammatory signalling on S100A14 levels. Thus, EMT/ZEB1 and IL-6/11-STAT3 act together to establish the expression pattern of S100 proteins that favours cell invasion.

## Methods

### Patients’ samples and immunohistochemistry

Immunostaining of PDAC series of samples (*n* = 31) was performed on 4-μm thick sections, serially cut from the paraffin blocks. Tissue microarrays (TMA) were purchased from US-Biomax (Rockville, MD, USA). The primary antibody/antigen complex was detected using the Novolink™ Polymer Detection System (Novocastra Laboratories, Newcastle upon Tyne, UK). All slide images were viewed and captured using Hamamatsu Slide Scanner microscope. The staining was performed in parallel with a negative control (no primary antibody added) to exclude any nonspecific background staining. Specificity of anti-S100A4, anti-S100A6 and anti-S100A14 antibodies was validated using cytoblocks prepared from PDAC cells, in which corresponding proteins were depleted using siRNAs. Evaluation of pancreatic pathology specimens was performed by two independent qualified pathologists. The intensity of staining was assessed for each section on a four-point scale: − = negative, + = low, ++ = moderate and +++ = intense staining.

### Cell lines, treatments, and transfections

Pancreatic cancer cell lines were obtained from the American Type Culture Collection (ATCC) and cultured in the 5% CO_2_ and 37 °C incubator in Roswell Park Memorial Institute medium supplemental with 10% FBS according to the ATCC recommendations. In some experiments, cells were treated 200 ng/ml of cytokines or chemokines and cultured for 48 h before harvesting. A431 or MCF7 cell lines with doxycycline-regulated expression of ZEB2 or ZEB1 were cultured in Dulbecco’s Modified Eagle’s medium supplemented with 10% foetal bovine serum (FBS) in the presence of absence of 1 μg/ml doxycycline. STAT3 inhibitor stattic (Sigma Aldrich, St Louis, MO, USA) was used at the concentration 5 μM. Proteasome inhibitor MG132 (Sigma Aldrich) was added at the increasing concentrations 16 h prior cell lysis. Cells were transfected by electroporation (a single pulse of 250V and 250 Fd by using the Gene Pulser Xcell electroporation system; BioRad Laboratories, Hercules, CA, USA) according to the manufacturer’s protocol.

### Plasmids and siRNAs

Construction of a vector expressing GFP-tagged ZEB1 was described previously.^17^ siRNA control, and siRNA targeting S100A4, S100A6, S100A11, S100A14, and STAT3 were purchased from Dharmacon (Lafayette, CO, USA), Sigma-Aldrich or Ambion (Austin, TX, USA) (see Supplementary Table S1).

### Immunoblotting

Cells were lysed in Laemmli buffer; lysates were normalised for equal protein concentrations, size fractionated in SDS-PAGE gels and subsequently transferred to the PVDF membranes (Millipore, Bedford, MA, USA). The membranes were blocked in 3% bovine serum albumin for 1 h, and then incubated in the primary antibodies either for 1 h or overnight at 4 °C. Next, the membranes were incubated with the horseradish peroxidase-conjugated secondary antibodies (1:2000; DAKO, Glostrup, Denmark) for 1 h. The protein bands were detected using enhanced chemiluminescence Western blotting substrate (Thermo Scientific, Waltham, MA, USA).

We used the following primary antibodies raised against ZEB1 and S100A8, (both from Santa Cruz Biotechnology, Dallas, USA); S100A4 and S100A6 (both from Proteintech, Manchester, UK); S100A2, S100A9, S100A14, S100P, Tubulin and β-Actin (all from Sigma Aldrich); SNAIL1, SNAIL2, STAT3, pSTAT3, p65 (Cell Signaling Technology, Danvers, MA, USA); E-cadherin and P-cadherin (BD Bioscience, San Jose, CA, USA); S100A11 (R&D Systems, Minneapolis, MN, USA); TWIST1 (Abcam Cambridge, MA, USA); ZEB2 (in-house made^18^). Tubulin or β-Actin staining was used to control equal loading.

### Quantitative PCR

Total RNA was isolated by the RNeasy RNA isolation kit (Qiagen, Germantown, MD, USA) and applied for the cDNA synthesis using RevertAid H Minus First Strand cDNA Synthesis Kit (Thermo Scientific). Quantitative PCR was carried out in triplicate experiments on Roche 480 Fast Real-Time PCR system using Fast SYBR Green Master Mix (Applied Biosystems, Warrington, UK) in 40 cycles with the annealing/extension temperature 60 °C. The quantitative data were normalised to an internal control (*GAPDH*). Dissociation curves were examined to exclude the risk of nonspecific amplification. Conventional ΔΔCT method was adopted to analyse the data. PCR primer sequences are shown in Supplementary Table S2.

### Immunofluorescence

Cells were cultured on coverslips, fixed in 4% paraformaldehyde, permeabilised in 0.5% Triton X-100, incubated with the primary anti-S100A14 antibody (Sigma Aldrich) for 1 h, and AlexaFluor 594-conjugated secondary antibody (Invitrogen, Grand Island, NY, USA). After counterstaining with DAPI (Molecular Probes, Invitrogen), cells were examined and photographed using a confocal microscope (Zeiss Axiovert 200 M).

### In vitro cell invasion assay

PDAC cells were transfected with siRNAs and seeded on 60 mm dishes. 24 h post-transfection, cells were counted, and 5 × 10^4^ (BxPC-3); 7 × 10^4^ (AsPc-1, MIA PaCa-2) or 10^5^ cells (SU.86.86) were resuspended in 100 μl RPMI medium and seeded on porous transwell membranes (pore size, 8 μm; BD Biosciences) coated with collagen I or fibronectin (Corning, NY, USA). Cells were allowed to migrate through collagen I barrier towards FBS gradients for 18 h. Invasion of MIA PaCa-2 cells through fibronectin-coated membranes was stopped and analysed in 6 h after seeding. Cells remaining at the upper surface of the membranes were removed using a cotton swab. The membranes were treated with methanol and stained with Gurr rapid staining kit (BDH, Poole, UK). The number of invaded cells was counted in five random fields using an inverted Nikon TE2000-U microscope.

### Zebrafish embryo invasion assay

Twenty four hpf embryos were dechorionated under a stereo-microscope, anaesthetised in 0.02% Tricaine solution (ethyl 3-aminobenzoate methane sulfonate, Sigma Aldrich) and immobilised in 1% low melting agarose. Cells were harvested, quantified, fluorescently labelled by incubating for 1 h with the DilC12 dye (2.5 μg/ml, Thermo Scientific) and extensively washed with PBS. In the experiments with IL-11, cells were treated with the cytokine for 48 h, or mock-treated. In the experiments with siRNAs, cells were transfected, seeded on 60 mm dishes, harvested 48 h post-transfection, labelled and washed. 1000 cells per fish were microinjected into the perivitelline cavity regions using a Pneumatic Injector set at a pressure of 500–1000 hPa and time of 0.3–0.8 s. Embryos were checked under fluorescent microscope one-hour post-injection (hpi), and those with fluorescent cells outside the desired injection region were excluded from the analysis. Next, the embryos were cut out from agarose using forceps, kept in water at 33 °C, and 48 hpi the fish were mounted in agarose again, and imaging was conducted by phase contrast combined with fluorescence microscopy using ×4 objective. Image montage and analysis of cell migration were carried out using ImageJ software. If fluorescence was detected in the vasculature and throughout the fish body, this was considered as an evidence of invasion. In the absence of invasion, fluorescence was localised exclusively to the perivitelline space and yolk sac.

### Statistics

In IHC experiments, all statistical analyses were performed using Statistical Package for the Social Sciences 20.0^®^ (SPSS, Chicago, Illinois, USA). Associations between different proteins were determined using the Pearson correlation coefficient. Results from this test produced a correlation coefficient, indicating the strength and direction of the association, and a *p*-value, indicating the significance. The Kruskal–Wallis test and Mann–Whitney *U*-test were used to compare continuous and ordinal variable between subgroups. Statistical significance was defined as *p* < 0.05.

To correlate expression of *S1004*, *S100A6* and *S100A14* genes with EMT markers in PDAC cell lines, data from Expression Atlas (CCLE cohort) were downloaded to the R software. Data were analysed using Pheatmap add-on to generate non-hierarchical clustering of the selected genes.

To compare invasive potentials of cells in zebrafish embryos statistical differences were determined using the Student’s *t*-test.

## Results

### EMT perturbs expression of S100 gene family in pancreatic carcinoma cells

**Fig. 1:**
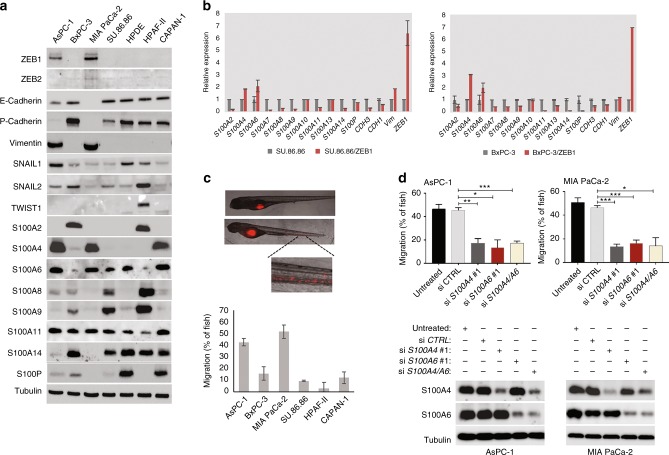
Expression of S100 family members is associated with EMT, and mesenchymal S100 proteins stimulate invasion of PDAC cells. **a** Immunoblot analysis of EMT-TFs, EMT markers and S100 proteins in a panel of PDAC cell lines. **b** Analysis of the transcription of ZEB1-regulated *S100* genes in epithelial PDAC cells. BxPC-3 and SU.86.86 cell lines were transfected with the plasmid vectors expressing GFP-tagged ZEB1 or GFP control and cultured for 48 h. Bar charts show the expression of genes encoding S100 proteins and EMT markers analysed by qPCR. Data represent the mean of three replicate experiments ± StDev. **c** Invasion of mesenchymal and epithelial PDAC cell lines in zebrafish embryos. Cells were fluorescently DilC12-labelled (red), microinjected into the perivitelline cavities of zebrafish embryos, imaged and analysed 48 hpi as described in Methods. Merged phase contrast and fluorescence images are representative examples of zebrafish embryos with no invasion (upper image) or with PDAC cells that intravasate into the circulation (lower image). Minimum 10 fish per cell line were used in each experiment. Results are mean ± StDev of three independent experiments. **d** Mesenchymal S100 proteins, S100A4 and S100A6, contribute to the enhanced invasive potential of PDAC cells in vivo. siRNA-mediated knockdowns of mesenchymal *S100* genes reduce the invasion of AsPC-1 and MIA PaCa-2 cells in zebrafish assay. Western blots show the extent of depletion of S100A4 and S100A6. Results are means (*n* = 3 biological replicates; 10 fish in each experiment) ± StDev. *p < 0.05; **p < 0.01; ***p < 0.001

The implication of individual S100 family members in EMT has been reported in several cancer types. However, to our knowledge, no effort has been made to address how an EMT affects expression of different family members in one study. To address this, we correlated the expression levels of S100 proteins with the EMT status in a panel of pancreatic carcinoma cell lines. Among five EMT-TFs (SNAIL1, SNAIL2, ZEB1, ZEB2 and TWIST1) analysed in these experiments, only ZEB1 correlated with enhanced vimentin expression, and reduced levels of E- and P-cadherins (Fig. 1a). Two cell lines, AsPC1 and MIA PaCa-2, exhibited mesenchymal expression patterns and expressed high levels of S100A4, S100A6 and S100A11. Most of the S100 proteins, however, were overrepresented in PDAC cell lines displaying epithelial characteristics, and the expression of S100A14 perfectly correlated with the presence of epithelial markers (Fig. 1a). The same correlations were observed on the transcriptional level, mesenchymal AsPC1 and MIA PaCa-2 cell lines expressed highest levels of *S100A4/6* but no *S100A14* mRNA (Supplementary Fig. S1). We extended this analysis by interrogating Cancer Cell Line Encyclopaedia (CCLE) gene expression dataset. Unsupervised clustering identified association of *S100A4*/*A6* genes with the mesenchymal marker *VIM* and *ZEB1*. *S100A14* clustered with the *CDH1* gene encoding E -cadherin (Supplementary Fig. S2).

To analyse whether an EMT is capable of altering the S100 expression profile in pancreatic cancer cells, we overexpressed ZEB1 in two epithelial PDAC cell lines, BxPC-3 and SU.86.86. In 96 h post-transfection, we observed a remarkable downregulation of E-cadherin and induction of vimentin indicating that both cell lines are responsive to ZEB1-induced EMT (Supplementary Fig. S3). In 48 h post-transfection, we performed qPCR analysis of *S100* transcriptomes in both cell lines. ZEB1-induced expression of only two genes, *S100A4* and *S100A6*, whereas transcription of eight genes (*S100A2*, *S100A7*, *S100A8*, *S100A9*, *S100A11*, *S100A14* and *S100P*) was repressed (Fig. 1b). Similarly, we found that most of these genes were downregulated by ZEB proteins in cellular models of EMT unrelated to PDAC, MCF7/ZEB1 and A431/ZEB2 (data not shown). Thus, we concluded that most of the *S100* genes are predominantly expressed in epithelial cell lines, and two family members, *S100A4* and *S100A6* can be categorised into a mesenchymal group. *S100A14* displayed features of an epithelial marker: it clustered with epithelial cadherins in PDAC cell lines and was strongly repressed by ZEB1 (Fig. 1a, b; see also Fig. 6).

### Mesenchymal S100 proteins are required for the enhanced invasion of mesenchymal pancreatic cancer cells in zebrafish xenograft models

S100A2, S100A4, S100A6, S100A7, S100A14 and S100P proteins are involved in the regulation of cell migration/invasion and cytoskeletal dynamics, i.e., biological processes representing hallmarks of EMT.^19^ We aimed to investigate whether perturbed expression of S100 family members is a part of a mechanism through which EMT activates cell invasion. We analysed the effect of depleting mesenchymal S100 proteins on the invasion of PDAC cells in vitro. Knockdown of *S100A4* or *S100A6* using two different siRNAs had no effect on cell viability (data not shown), but significantly reduced invasion of AsPC1 cells through collagen I-coated membranes in transwell assays (Supplementary Fig. S4). MIA PaCa-2 cells lack collagen I receptor integrin α2β1 and do not adhere to this substrate.^20^ However, knockdown of either of the mesenchymal *S100* genes decreased invasion of these cells through a layer of fibronectin more than twofold (Supplementary Fig. S4). Conversely, S100A4 or S100A6 depletion in a unique epithelial cell line expressing these proteins, CAPAN-1, produced no effect on cell invasion in vitro (data not shown).

Next, we employed zebrafish embryo xenotransplantation model to analyse invasive capabilities of PDAC cells in vivo. Zebrafish embryo invasion assay has been broadly used to study invasion of PDAC cells and early stages of tumour metastasis in the past.^21,22^ As the immune system in zebrafish embryos is immature, there is no rejection of human xenografts. Optical transparency of the embryos allows visualisation of injected fluorescently-labelled cells. Additionally, relative simplicity of the methodology allows simultaneous examination of many embryos improving the validity of the statistical analysis. Expectedly, mesenchymal cell lines AsPC1 and MIA PaCa-2 displayed enhanced invasive capabilities as compared to the epithelial PDAC cells (Fig. 1c). Depletion of either S100A4 or S100A6 by siRNA strongly reduced invasive capacity of both AsPC1 and MIA PaCa-2 cell lines in vivo (Fig. 1d), whereas depletion of S100A11 had no effect (Supplementary Fig. S5). S100A11 is ubiquitously expressed in pancreatic cancer cell lines and show no correlation with the differentiation status of PDAC cells (Fig. 1a, and Supplementary Fig. S1). Of note, combined depletion of both S100A4 and S100A6 produced the same effect on cell invasion as individual knockdowns suggesting that these two proteins do not functionally compensate for each other (Fig. 1d).

### S100A14 is an epithelial marker repressing cell invasion

**Fig. 2:**
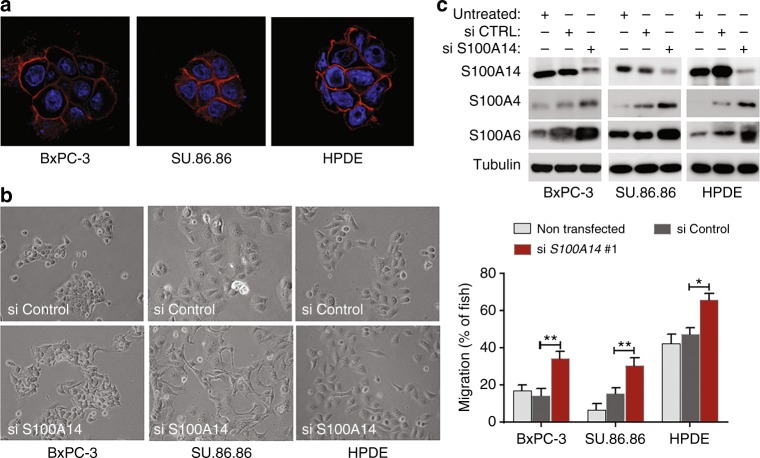
S100A14 sustains epithelial morphology and suppresses cell invasion. **a** Subcellular localisation of S100A14 was examined in BxPC-3 and SU.86.86 epithelial PDAC cell lines, and in human pancreatic duct epithelial (HPDE) cells by immunofluorescence. Nuclei are stained in blue (DAPI). **b** Phase-contrast images of PDAC epithelial cells transfected with S100A14-targeting or control siRNAs. *S100A14* knockdown results in cell scattering. **c** Depletion of S100A14 stimulates invasion of epithelial PDAC cells in zebrafish embryos. Bars represent means (*n* = 3 biological replicates; 10 fish in each experiment)+/− StDev. *p < 0.05; **p < 0.01 (Student’s *t*-Test). Western blots illustrate the degree of S100A14 depletion. Knockdown of *S100A14* enhances expression levels of mesenchymal S100 proteins, but not canonical EMT markers, E-cadherin and vimentin (data not shown)

S100A14 is an epithelial protein, expressed exclusively in epithelial pancreatic cancer cell lines, and repressed by ZEB1. Reduction in S100A14 expression was also reported in A549 lung cancer cells undergoing an EMT in response to TGFβ1 treatment (GEO GSE17708) and S100A14 clustered with epithelial markers in breast cancer cells.^23^ In epithelial PDAC cells, S100A14 is localised to the cell–cell contacts indicating its potential role in maintenance of the epithelial morphology (Fig. 2a). Indeed, siRNA-mediated *S100A14* knockdown resulted in morphological EMT (Fig. 2b) and an increase in the expression levels of mesenchymal S100 proteins (Fig. 2c), but the canonical EMT markers were not affected (data not shown). Knockdown of *S100A14* with two unrelated siRNAs in BxPC-3 or SU.86.86 cell lines produced no effect on cell viability (data not shown), but significantly activated cell invasion through collagen barrier (Fig. S6). Furthermore, in accordance with these results, S100A14 depletion significantly activated in vivo invasion of epithelial cells (Fig. 2c).

### Immunodetection of S100A4/S100A6 and S100A14 proteins in PDAC samples: correlation with the EMT status of the tumours

**Fig. 3:**
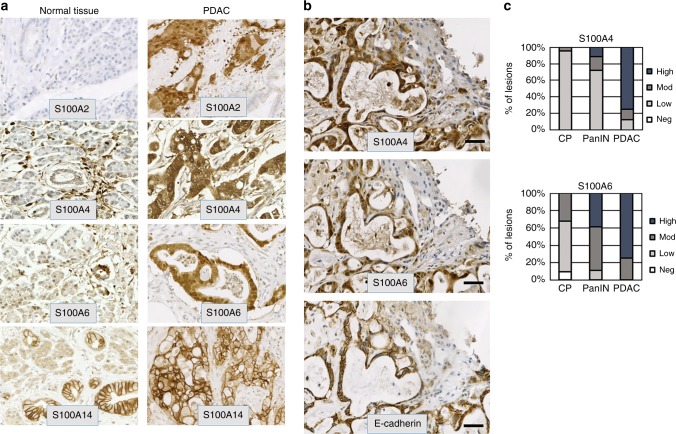
S100 proteins are expressed in pancreatic lesions. **a** Examples of IHC analyses of the expression of S100 proteins in a series of PDAC samples (*n* = 31). **b** Images exemplify co-expression of mesenchymal S100 proteins and E-caherin in parallel sections of a PDAC sample. **c** Bar charts illustrate the frequency of the expression of S100A4 and S100A6 proteins in different types of pancreatic lesions presented on TMA

To validate our in vitro observations, we employed a series of PDAC samples (*n* = 31) to examine the expression of mesenchymal proteins (S100A4 and S100A6) and two proteins detected exclusively in the epithelial cell lines, S100A14 and S100A2 (Fig. 1a). Normal acinar and ductal cells expressed no or very low levels of S100A4, S100A6, or S100A2 but were positive for S100A14 (Fig. 3a). 61, 83 and 83% of tumours showed moderate to strong staining for S100A4, S100A6 and S100A14, respectively, whereas only 22% were positive for S100A2. The expression of S100A2, S100A4 and S100A6 were detected in the cytoplasm and/or nuclei (Fig. 3a). In contrast, S100A14 exhibited predominantly membranous staining in accordance with the results of the immunofluorescence analysis in vitro (see Fig. 2a). While positive and negative correlations of S100A14 expression with E-cadherin and vimentin, respectively were extremely significant (*p* < 0.0001), we observed no correlation between S100A2 and other markers (Table 1). Expressions of S100A4 and S100A6 significantly correlated with each other (*p* = 0.012); and S100A4 positively correlated with ZEB1 (*p* = 0.016) and, consistent with a previous report,^24^ negatively with E-cadherin (*p* = 0.022) (Table 1). Although significant, this correlation was far from being perfect. Remarkably, out of 18 specimens scored as strongly positive for E-cadherin expression, 6 were also strongly positive for S100A4 (Fig. 3b). We interpreted this observation as an indication that EMT is not the only mechanism responsible for the upregulation of mesenchymal S100 proteins in pancreatic cancer cells.

**Table 1:** Association between EMT markers and expression of S100 proteins in PDAC samples

		S100A2	S100A4	S100A6	S100A14	Vimentin	P-Cad	E-Cad	Slug	Twist	ZEB1	pSTAT3
S100A2	Pearson Correlation	1	0.246	.047	.121	−.001	0.173	0.170	0.030	0.057	0.301	−0.091
	Sig. (2-tailed)		0.181	0.801	0.517	0.994	0.351	0.362	0.872	0.760	0.100	0.627
	*N*	31	31	31	31	31	31	31	31	31	31	31
S100A4	Pearson Correlation	0.246	1	0.444*	−0.371*	0.042	0.074	−0.409*	0.207	0.310	0.428*	0.470**
	Sig. (2-tailed)	0.181		0.012	0.040	0.821	0.692	0.022	0.263	0.090	0.016	0.008
	*N*	31	31	31	31	31	31	31	31	31	31	31
S100A6	Pearson Correlation	0.047	0.444*	1	0.096	−0.007	−0.030	0.012	0.156	0.189	0.083	0.387*
	Sig. (2-tailed)	0.801	0.012		0.607	0.970	0.873	0.948	0.403	0.310	0.657	0.032
	*N*	31	31	31	31	31	31	31	31	31	31	31
S100A14	Pearson Correlation	0.121	−0.371*	0.096	1	−0.589***	0.338	0.828***	−0.088	−0.221	−0.264	−0.261
	Sig. (2-tailed)	0.517	0.040	0.607		0.000	0.063	0.000	0.636	0.232	0.152	0.155
	*N*	31	31	31	31	31	31	31	31	31	31	31
Vimentin	Pearson Correlation	−0.001	0.042	−0.007	−0.589***	1	−0.180	−0.422*	0.069	0.237	−0.035	0.283
	Sig. (2-tailed)	0.994	0.821	0.970	0.000		0.332	0.018	0.712	0.199	0.854	0.124
	*N*	31	31	31	31	31	31	31	31	31	31	31
P-Cad.	Pearson Correlation	0.173	0.074	−0.030	0.338	−0.180	1	0.426*	0.210	0.042	0.369*	−0.077
	Sig. (2-tailed)	0.351	0.692	0.873	0.063	0.332		0.017	0.257	0.823	0.041	0.680
	*N*	31	31	31	31	31	31	31	31	31	31	31
E-Cad.	Pearson Correlation	0.170	−0.409*	0.012	0.828***	−0.422*	0.426*	1	−0.203	−0.318	−0.176	−0.296
	Sig. (2-tailed)	0.362	0.022	0.948	0.000	0.018	0.017		0.273	0.081	0.345	0.106
	*N*	31	31	31	31	31	31	31	31	31	31	31
Slug	Pearson Correlation	0.030	0.207	0.156	−0.088	0.069	0.210	−0.203	1	0.233	0.310	0.056
	Sig. (2-tailed)	0.872	0.263	0.403	0.636	0.712	0.257	0.273		0.206	0.090	0.765
	*N*	31	31	31	31	31	31	31	31	31	31	31
Twist	Pearson Correlation	0.057	0.310	0.189	−0.221	0.237	0.042	−0.318	0.233	1	0.125	0.419*
	Sig. (2-tailed)	0.760	0.090	0.310	0.232	0.199	0.823	0.081	0.206		0.505	0.019
	*N*	31	31	31	31	31	31	31	31	31	31	31
ZEB1	Pearson Correlation	0.301	0.428*	0.083	−0.264	−0.035	0.369*	−0.176	0.310	0.125	1	0.122
	Sig. (2-tailed)	0.100	0.016	0.657	0.152	0.854	0.041	0.345	0.090	0.505		0.512
	*N*	31	31	31	31	31	31	31	31	31	31	31
p-STAT3	Pearson Correlation	−0.091	0.470**	0.387*	−0.261	0.283	−0.077	−0.296	0.056	0.419*	0.122	1
	Sig. (2-tailed)	0.627	0.008	0.032	0.155	0.124	0.680	0.106	0.765	0.019	0.512	
	*N*	31	31	31	31	31	31	31	31	31	31	31

Asterisks indicate significant positive and negative correlations. *p < 0.05; **p < 0.01; ***p < 0.0001

### Mesenchymal S100 proteins are expressed in chronic pancreatitis (CP), pancreatic intraepithelial neoplasia (PanIN) and PDAC albeit at different levels

As S100A4 and S100A6 proteins were detected in a subset of well-differentiated PDAC, we hypothesised that their activation occurs in early malignant or premalignant pancreatic lesions. To test whether expression of S100A4 and S100A6 proteins is detectable in early lesions, we applied a commercial tissue microarray containing CP (*n* = 22), PanIN (*n* = 18), and PDAC (*n* = 8) samples. Both proteins were present in the majority of CP and PanIN samples, but the expression was either mostly weak (S100A4) or weak-to-moderate (S100A6). Immunopositivity for both S100A4 and S100A6 increased sequentially from CP to PanIN to PDAC (*p* < 0.001 for both markers; Fig. 3c, Supplementary Tables S3 and S4).

### Cytokine/STAT3 signalling regulates expression of S100 proteins in pancreatic cancer

**Fig. 4:**
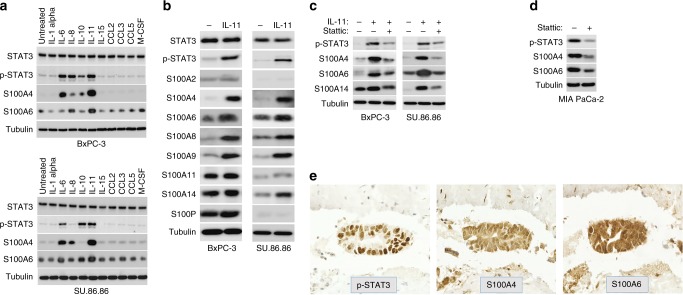
IL-6/11-STAT3 signalling regulates expression of S100 proteins in PDAC cells. **a** IL-6 and IL-11 enhance expression levels of S100A4 and S100A6 proteins in PDAC cell lines. PDAC cells were exposed to the specified cytokines (200 ng/ml) for 48 h, and changes in protein expression levels were assessed by immunoblotting with indicated antibodies. **b** IL-11 enhances expression of S100 proteins belonging to both mesenchymal and epithelial groups. BxPC-3 and SU.86.86 cells were cultured in the presence of IL-11 for 48 h, and protein expression was analysed by immunoblotting. **c** STAT3 mediates the effect of IL-11 on the expression of S100 proteins in epithelial PDAC cell lines. Expression of S100 proteins was stimulated by IL-11 treatment for 48 h. A selective STAT3 inhibitor stattic was added 24 h before cells were harvested, and expression of S100 proteins was analysed by immunoblotting. **d** STAT3 controls expression of S100 proteins in mesenchymal cells, where their expression is intrinsically high. MIA PaCa-2 cells were treated with stattic for 24 h prior the immunoblot analysis with indicated antibodies was carried out. **e** Parallel sections from the same block containing a PDAC tissue were analysed by IHC. The images exemplify samples, in which S100A4, S100A6 and pSTAT3 proteins are co-expressed

Inflammation is commonly correlated with the initiation and development of PDAC, and CP is a prerequisite for the initiation of K-Ras-induced PDAC in a mouse model.^25^ The presence of mesenchymal S100 proteins in CP, PanIN and PDAC was compatible with the hypothesis that inflammatory pathways regulate their expression in pancreatic lesions. To test this, we stimulated two epithelial PDAC cell lines, BxPC-3 and SU.86.86, with a series of selected recombinant human cytokines and chemokines including IL-1α, IL-6, IL-8, IL-10, IL-11, IL-15, CCL-2, CCL-3, CLL-5 and M-CSF. The effect of these treatments on the expression of S100A4 and S100A6 was analysed in 48 h. We found that two phylogenetically related cytokines, IL-11 and IL-6, activated expression of both mesenchymal S100 proteins more efficiently than other stimuli. Whereas IL-11 induced expression of both mesenchymal S100 proteins in both cell lines, IL-6 effectively induced S100A4 in BxPC-3 and SU.86.86 cell lines, and S100A6 in SU.86.86 cells (Fig. 4a, b). Upregulation of both genes occurred at the transcriptional level (Supplementary Fig. S7). Of note, expression of epithelial S100 proteins, such as S100A8, S100A9 and S100A14 was also activated upon IL-11 treatment (Fig. 4b, see also Figs. 4c and 6c, and Supplementary Fig. S7).

IL-6 and IL-11 activate signal transducer and activator of transcription 3 (STAT3), and this canonical pathway may represent a mechanism by which chronic inflammation contributes to tumour initiation and progression. Consistently, phosphorylated STAT3 was detected in the lysates of cells treated with IL-6 or IL-11 (Fig. 4a, b). To analyse STAT3 function in IL-mediated regulation of S100 proteins we made use of stattic, a specific small-molecule inhibitor of STAT3 activation and dimerisation. Treatment with stattic diminished the IL-11-mediated increase in the expression levels of S100A4, S100A6 and S100A14 proteins (Fig. 4c). In agreement with this observation, stattic strongly decreased the levels of S100A4, and S100A6 proteins also in MIA PaCa-2 cells, where both proteins are highly abundant (Fig. 4d). Although *S100A4* and *S100A6* were reported to be NF-κB target genes,^26,27^ and NF-κB is implicated in PDAC pathogenesis,^28^ this signalling played no role in upregulation of S100 proteins in PDAC cells (Supplementary Fig. S8).

To address whether our observation is relevant to the activation of S100A6 and S100A4 in tumours, we assessed the expression of pSTAT3 in PDAC specimens by IHC. Remarkably, pSTAT3 immunopositivity significantly correlated with the expression of S100A4 (*p* = 0.008) and S100A6 (*p* = 0.032), but not with that of the epithelial protein S100A14 or S100A2 (Table 1, and Fig. 4e).

### S100A4 and S100A6 are determinants of cytokine-STAT3-induced invasion of PDAC cells

**Fig. 5:**
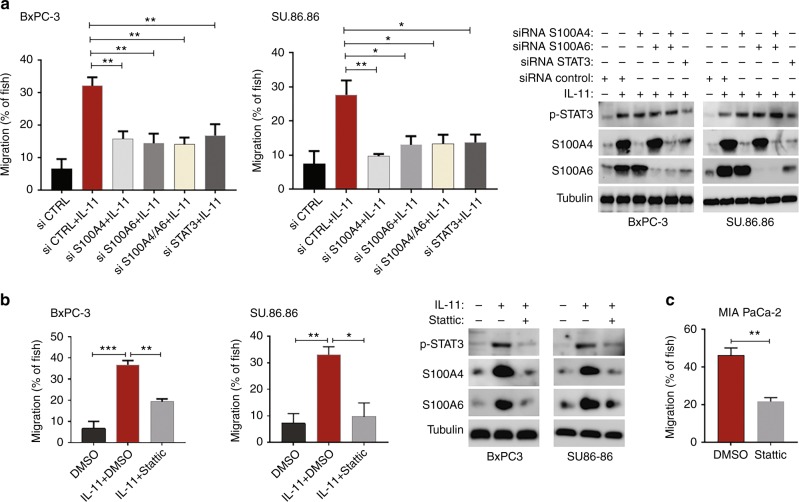
Mesenchymal S100 proteins mediate IL-11/STAT3-driven invasion in zebrafish embryos. **a** IL-11 stimulates invasion of PDAC cells in STAT3-dependent manner. Epithelial PDAC cells were treated with the control siRNA, or siRNA targeting S100A4, S100A6, or STAT3, cultured in the presence or absence of IL-11 for 48 h, and analysed in zebrafish invasion assay. Expression of STAT3 and S100 proteins was analysed by immunoblotting. **b** BxPC-3 and SU.86.86 cells were treated with IL-11 alone or in combination with stattic, and their invasiveness was assessed in zebrafish embryos. **c** Mesenchymal MIA PaCa-2 cells were treated with stattic, or mock-treated, and invasion analysed in zebrafish embryos. **a**–**c** Results are mean ± StDev of biological replicates (*n* = 3). Cell invasion was analysed in 10 fish in each experiment. *p < 0.05; **p < 0.01; ***p < 0.001 (Student’s *t*-Test)

IL-6-type cytokines promote invasive ability in carcinoma cells including PDAC.^29,30^ In agreement with numerous reports implicating STAT3 signalling in invasiveness, treatment of BxPC-3 and SU.86.86 cells with IL-11 strongly activated cell invasion in zebrafish embryos. The effect of IL-11 was largely dependent on STAT3 activation, because invasion of IL-11-treated cells was inhibited by siRNA specific for STAT3 (Fig. 5a) or by stattic (Fig. 5b). Likewise, stattic significantly reduced invasion of MIA PaCa-2 cells, in which the STAT3 pathway was constitutively active (Fig. 5c). Application of STAT3-targeting siRNA reduced expression of S100A4/6 proteins in IL-11-stimulated cells and supported our conclusion that interleukins induced expression of S100 proteins via STAT3 (see Fig. 5a). Remarkably, siRNA-mediated reduction in the expression of mesenchymal S100 proteins nearly blocked the stimulatory effect of IL-11 on cell invasion, and the effect of S100A4 or S100A6 knockdown on cell invasion was similar to that produced by the depletion of STAT3 (Fig. 5a).

### STAT3 and EMT pathways act in parallel to define the expression pattern of S100 genes in PDAC

**Fig. 6:**
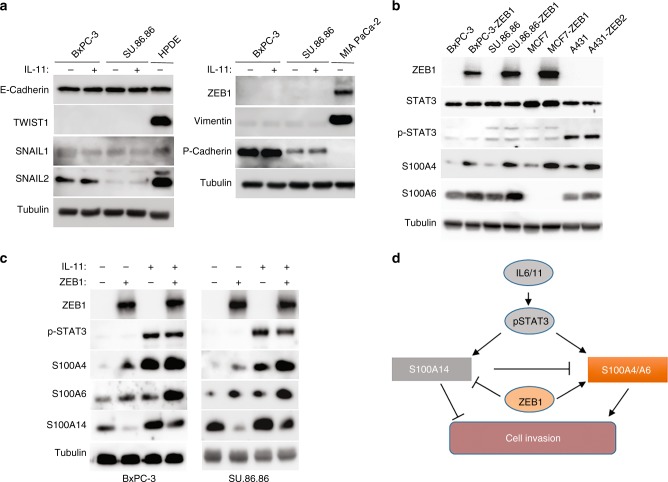
IL-11/STAT3 and ZEB1 regulate expression of S100 proteins in a mutually independent mode. **a** Treatment with IL-11 does not induce an EMT in epithelial PDAC cells. BxPC-3 and SU.86.86 cells were treated with IL-11 or mock-treated and the expression of EMT-TFs and EMT markers was analysed by immunoblotting as indicated. **b** Ectopic expression of ZEB proteins does not affect STAT3 phosphorylation. Effect of ZEB proteins on S100A4, S100A6 and pSTAT3 expression levels was analysed in indicated epithelial cell lines. Note that ZEB proteins regulate S100A4 and S100A6 independently of STAT3. (**c**) Combined effects of ZEB1 and IL-11 on the expression of S100 proteins. ZEB1-expressing or non-expressing cells were treated with IL-11 or left untreated, and level of the indicated S100 proteins was analysed by immunoblotting. **d** Schematic representation of EMT-ZEB1 and IL-11/6-STAT3 pathways regulating expression of mesenchymal (S100A4 and S100A6) and an epithelial (S100A14) S100 proteins in PDAC cells. ZEB1 cooperates with the inflammatory signalling to induce S100A4 and S100A6. On the other hand, it abolishes an effect of IL-11/IL-6 on the expression of S100A14. Reduced expression of S100A14 further stimulates production of S100A4 and S100A6. This regulatory network drives the invasion of PDAC cells

Reciprocal regulation of JAK/STAT and EMT pathways was reported in different carcinoma types.^31^ Therefore, we aimed to investigate whether ZEB1 and the IL-6/11-STAT3 module are parts of the same signalling pathway, or they act in parallel to modulate expression of S100 proteins and activate cell invasion. To this end, we analysed the effect of IL-11 on EMT status in epithelial PDAC cells. 48 h treatment was sufficient to induce expression of S100A4 and S100A6 proteins in both cell lines, BxPC-3 and SU.86.86, but no changes in the expression of EMT markers (Fig. 6a) or alterations in cell morphology (data not shown) was detected. Ectopic expression of ZEB1 enhanced the levels of S100A4 and S100A6, but this was independent of STAT3 activation (Fig. 6b). Importantly, these observations are in line with the lack of the correlation between ZEB1 and pSTAT3 in the series of PDAC samples (Table 1).

As no positive correlation between pSTAT3 and S100A14 was observed in PDAC specimens (Table 1), we speculated that certain oncogenic events uncouple S100A14 expression from STAT3 activation during pancreatic tumorigenesis. As ZEB1 acts independently of IL-6/11-STAT3 and down-regulates S100A14 (Fig. 1b), we proposed that it is a good candidate for a role of such a factor. Indeed, ectopic expression of ZEB1 reduced IL-11-mediated activation of S100A14 nearly to the steady-state level. In contrast, IL-11 and ZEB1 synergistically induced expression of both mesenchymal proteins, S100A4 and S100A6 in epithelial PDAC cells (Fig. 6c).

Overall, these data indicate that EMT and STAT3 do not constitute the same signalling pathway, but rather act in parallel to define the expression pattern of S100 family members leading to a highly invasive phenotype of PDAC cells (Fig. 6d).

## Discussion

S100 proteins have no intrinsic enzymatic activity and exert biological functions by modulating functions of their direct intracellular or extracellular targets. Global events in cell physiology such as oncogenic transformation, EMT or response to inflammation are associated with alterations in numerous signalling pathways and include changes in the expression pattern of the *S100* gene cluster. The relevance of this cluster to tumour biology has been demonstrated in many reports.^11^ In particular, a recent work has shown that amplification of the chromosome 1q21.3 region bearing the *S100* genes is associated with stem cell-like features, early relapse in breast cancer patients and chemotherapy resistance.^32^ These characteristics of aggressive tumours, as well as the ability of tumour cells to invade surrounding tissues, are hallmarks of EMT.

Most of the S100 proteins have been considered as EMT facilitators or EMT markers in certain carcinoma cell lines.^14^ S100A4 seems to represent a unique member of this protein family as its expression is universally used to detect EMT or EMT-like processes in various settings. Moreover, recent work has shown that S100A4 acts upstream of EMT-TFs in glioblastoma and is a master regulator of EMT-like events in this cancer type.^33^ Our study has shown that S100A4 and also S100A6, a family member displaying the highest homology with S100A4 (46% of identity and 59% of similarity), are indeed activated in the course of the ZEB1-induced EMT in PDAC cell lines. In agreement with this observation, expression of S100A4 was detected in most of ZEB1-expressing PDAC cell lines (Fig. 1a, S1 and S2), and correlated with ZEB1 expression in PDAC samples (Table 1). Of note, neither in tumour samples nor in the cell lines could we detect a correlation between S100 proteins and the other EMT-TFs, ZEB2, TWIST1 or SNAIL2.

Consistent with the enhanced expression of S100A4 and S100A6 in EMT, their presence (but not the presence of S100A11 or S100A14) in PDAC cells was a determinant of the increased invasion in vitro and in vivo. S100A4 physically interacts with the heavy chain of non-muscle myosin IIA, a major chemomechanical protein responsible for force generation in moving cells,^34–36^ and this interaction regulates formation of protrusions in the course of cell migration.^13^ Furthermore, S100A4 may stimulate cell migration and invasion via its association with rhotekin, a scaffold protein implicated in Rho signalling.^37^ The effect of S100A4 on cell motility is not necessarily limited to interactions within the cells. Indeed, treatment of cells with recombinant S100A4 may promote their motility, via interactions with growth factors or cytokines in extracellular milieu^38^ or via RAGE pathway stimulation.^39^ S100A6 was also shown to stimulate in vitro migration of tumour cells, including PDAC, but mechanistic understanding is still missing.^40,41^

The IHC analysis of PDAC specimens has shown that some of the samples retaining epithelial morphology expressed S100A4 and S100A6. This observation was in line with the assumption that other mechanisms, such as inflammation-activated pathways, regulate expression of S100A4 and S100A6 proteins at early stages of pancreatic tumorigenesis. Indeed, we detected both proteins in ductal cells in the CP and PanIN tissue samples, although at a significantly lower level than in PDAC specimens (Fig. 3c). In PanIN and PDAC, inflammatory signalling is amplified via a positive feedback mechanism. The driver mutation in the *K-Ras* oncogene induces production of IL-6 family cytokines or IL-1α leading to the autocrine activation of STAT3 or NF-κB pathways, and recruitment of the immune cells. The recruited cells, mostly of myeloid lineage, secrete IL-6, IL-1α and TNFα to further stimulate STAT3 and NF-κB in a paracrine manner,^42,43^ and these K-Ras-initiated pathways cooperate to promote the development of PDAC.^44^ Thus, inflammatory pathways mutually activated in cancer and myeloid cells via tumour-microenvironment crosstalk drive invasion and tumour spread.^45,46^ We established in vitro that S100A4 and S100A6 are targets of the IL6/IL11-STAT3 pathway, and found that their expression correlates with the presence of pSTAT3 in PDAC samples (Table 1). Therefore, we speculate that the amplification of the IL-6/11-STAT3 signalling during pancreatic tumorigenesis is a factor contributing to the gradual increase in the expression of mesenchymal S100 proteins in the CP-PanIN-PDAC sequence.

An interplay between the IL-6/11-STAT3 pathway and EMT has been extensively studied in different cancer types. In breast cancer cells, IL-6 dramatically induced the expression of TWIST1 and SNAIL1 via STAT3 activation, leading to the initiation of an EMT. TWIST1, in turn, stimulated production of soluble IL-6, thereby generating a positive feedback loop that maintained the mesenchymal phenotype and constitutively active STAT3 signalling.^46^ Likewise, in colorectal cancer, pSTAT3 promoted an EMT by direct transcriptional activation of the *ZEB1* gene.^47^ This observation is, however, in stark contrast to the studies demonstrating that in other colorectal carcinoma cell and mouse models, pSTAT3 inhibited EMT by destabilising SNAIL1 via the GSK-3β pathway.^48,49^ In accordance with the latter finding, STAT3 is capable of antagonising TGFβ-induced EMT in hepatocellular carcinoma cells through direct interaction with SMAD4.^50^ Therefore, as inflammation and EMT may exert reciprocal effects on each other, we proposed that ZEB1 and IL-6/11-STAT3 signalling belong to the same molecular pathway in PDAC. Contrary to our expectations, we could not find any interdependence between IL-11-STAT3 module and ZEB1-induced EMT in PDAC cell lines (Fig. 6a, b). Moreover, IHC examination of a series of PDAC samples revealed no correlation between pSTAT3 and EMT markers (E- and P-cadherins and vimentin) or ZEB1 (Table 1). However, IL-11-STAT3 and ZEB1 synergistically enhanced expression levels of S100A4 and S100A6 proteins, and pSTAT3 presence significantly correlated with S100A4 and S100A6 immunopositivity in pancreatic lesions. Thus, because IL-11-STAT3 signalling is a strong EMT-independent modulator of the expression of these genes, our data challenge the common view on S100A4 protein as a universal marker of EMT in PDAC.

IL-6/11-STAT3 signalling upregulated the expression of *S100* genes down-regulated by ZEB1. S100A14 was of particular interest, because this protein showed the features of a classical epithelial marker. Indeed, S100A14 expression extremely significantly correlated with EMT markers in PDAC samples (Table 1), its subcellular localisation was reminiscent of the distribution of proteins implicated in cell-cell adhesion in epithelial tissues, and S100A14 depletion activated cell invasion. The positive effect of IL-11 on S100A14 expression was neutralised by ZEB1, and therefore we propose a scheme whereby two independent pathways, IL-6/11-STAT3 and ZEB1, converge to stimulate expression of mesenchymal S100 proteins without increasing the expression of S100A14, which leads to the enhanced invasiveness of PDAC cells (Fig. 6d).

Our data imply that different S100 proteins may promote or repress invasion of PDAC cells. Development of selective inhibitors targeting the interactions of particular family members may have potential for clinical application in PDAC patients.

## Supplementary information

Supplementary files

## Data Availability

All data generated or analysed during this study are included in this published article and its supplementary information files.
