# Supplementary material for: ZEB1 and IL-6/11-STAT3 signalling cooperate to define invasive potential of pancreatic cancer cells via differential regulation of the expression of S100 proteins
Source: Br J Cancer. 2019 May 24;121(1):65–75. doi: 10.1038/s41416-019-0483-9 (PMC6738112; doi:10.1038/s41416-019-0483-9)
Supplement: Supplementary file 1 — Supplementary files [file 41416_2019_483_MOESM1_ESM.pdf]

EMT and IL6/11-STAT3 signalling pathway define expression patterns of S100 proteins and invasive potential of pancreatic cancer cells

Al-Ismaeel Q, Neal CP, Al-Mahmoodi HCS, Almutairi Z, Al-Shamarti I, Straatman K, Jaunbocus MN, Irvine A, Issa E, Moreman C, Dennison AR, Sayan AE, McDearmid J, Greaves P, Tulchinsky E, and Kriajevska M.

Supplementary material (Supplementary Figures and Tables)

Supplementary Figure S1

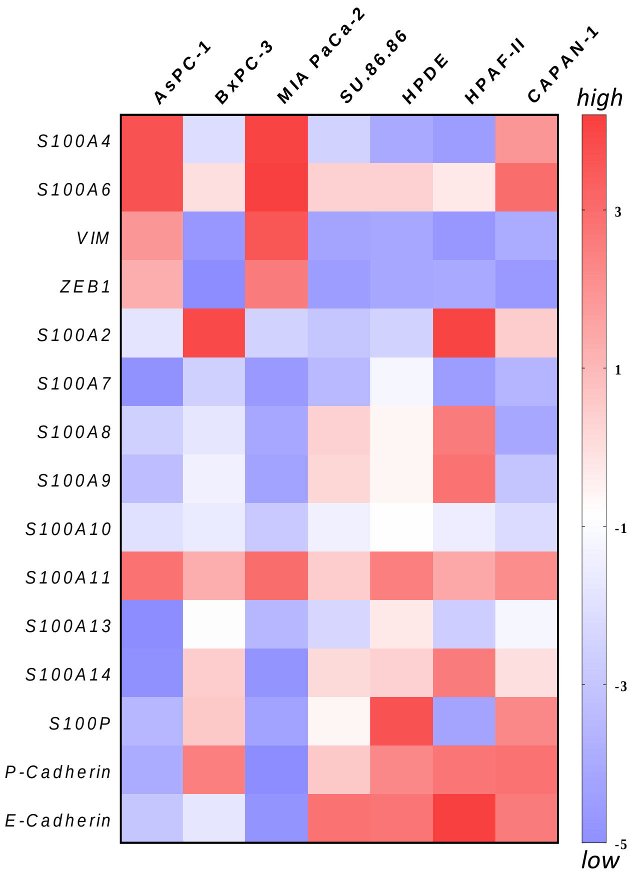

Supplementary Figure S1. Heatmap representation of qPCR analysis of the expression of EMT-associated genes and *S100* family members in indicated PDAC cell lines. *S100* genes were clustered based on their correlation with the expression of *ZEB1* and *VIM*.

Supplementary Figure S2

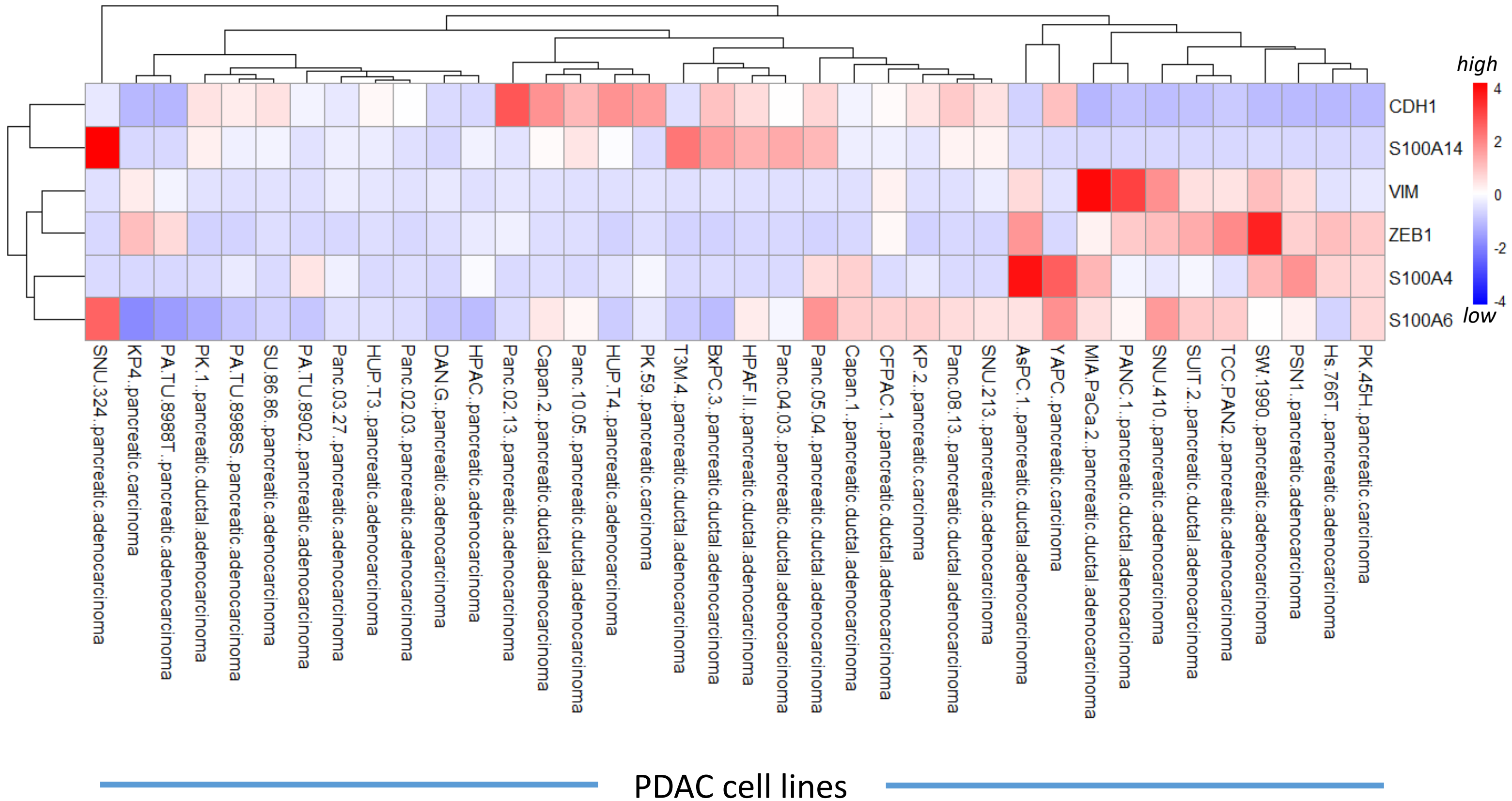

Supplementary Figure S2. Heatmap representation of non-hierarchical clustering of *S100A4*, *S100A6*, *S100A14*, *CDH1*, *ZEB1* and *VIM* gene expression in CCLE collection of PDAC cell lines (n=38). Red indicates high expression and blue indicates low expression. Note that *S100A14* and *S100A4/6* cluster with *CDH1* and mesenchymal markers respectively.

Supplementary Figure S3

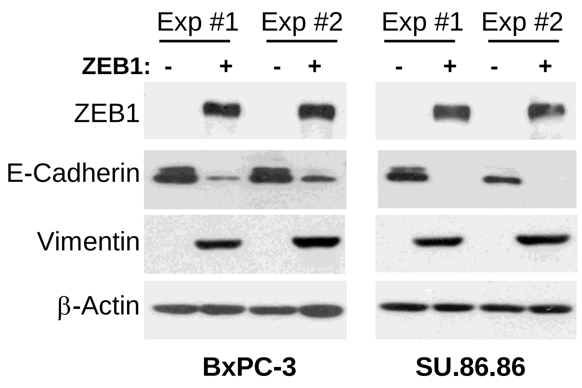

Supplementary Figure S3. ZEB1 activates EMT programs in BxPC-3 and SU.86.86 cell lines. Cells were transfected with a ZEB1-expressing vector and cultured at low density for 96 hours. Immunoblotting analysis shows repression of E-cadherin and activation of vimentin in two biological replicates.

Supplementary Figure S4

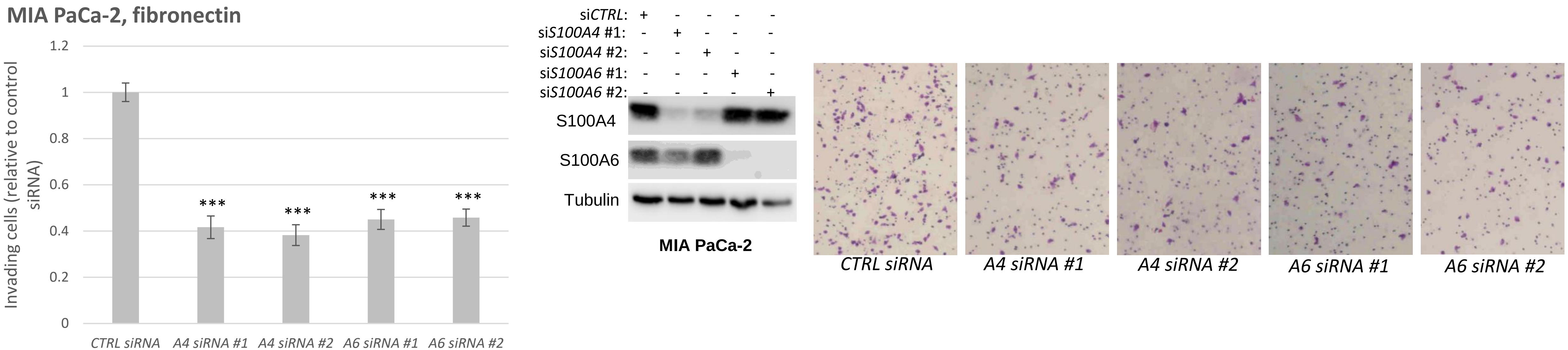

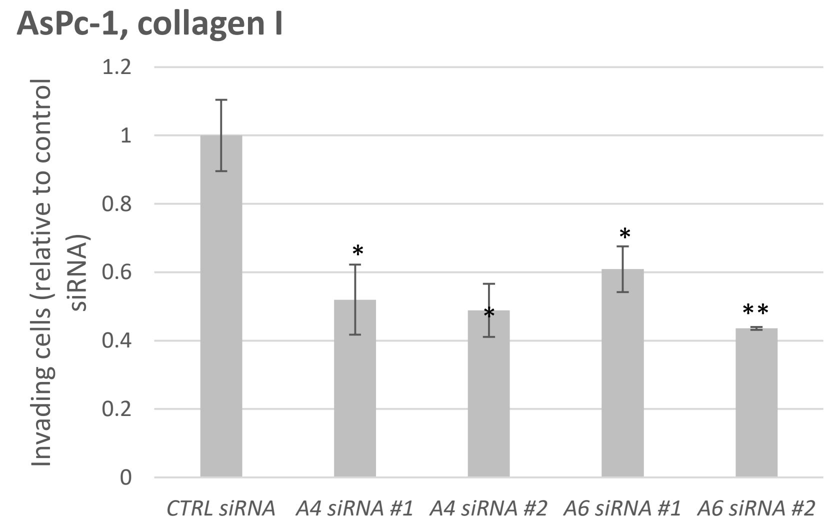

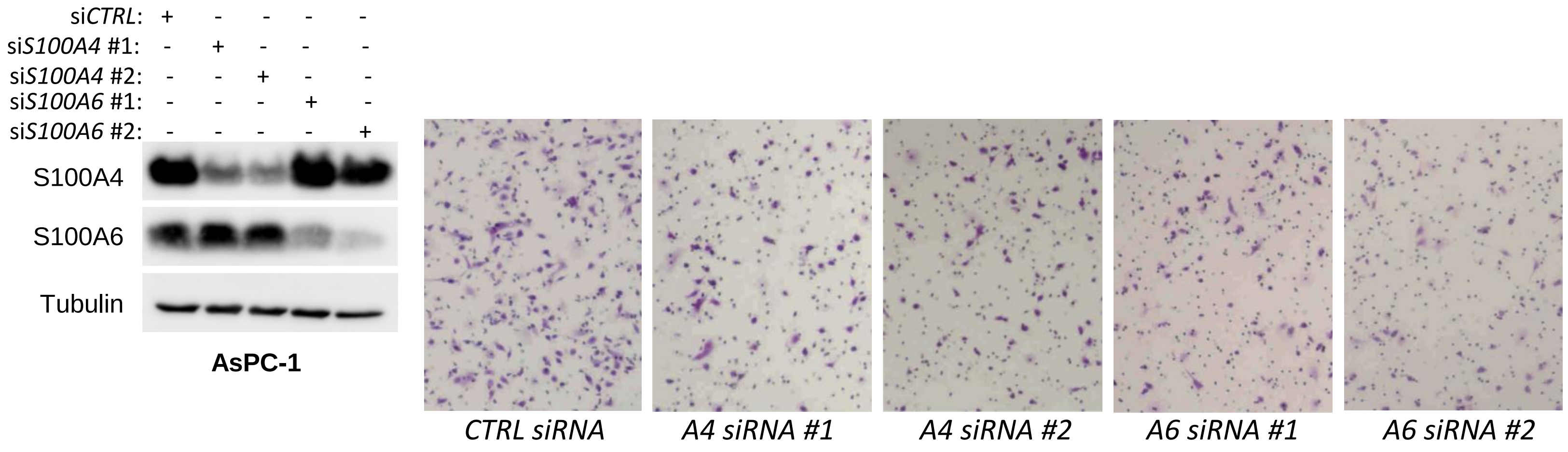

Supplementary Figure S4. S100A4 and S100A6 stimulate *in vitro* invasion of PDAC cells. MIA PaCa-2 or AsPC-1 cells were transfected with siRNAs targeting S100A4 or S100A6, or with control siRNA and seeded in Transwell inserts coated with collagen I or fibronectin. Cell invasion was analysed as described in Materials and Methods. Results are mean +/- StDev of triplicate experiments. *p<0.05; **p<0.01; ***p<0.001 (Student's t Test). The representative microscopy images of invaded cells are shown.

Supplementary Figure S5

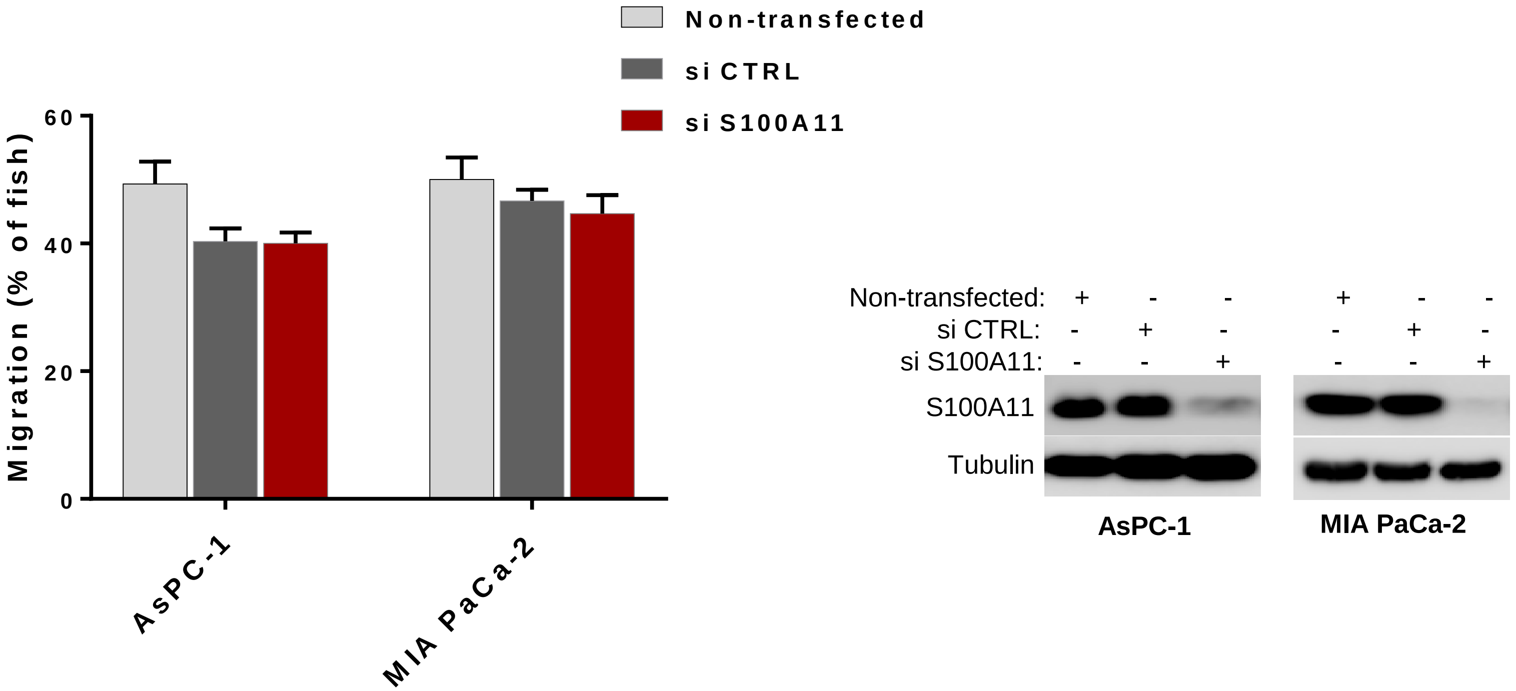

Supplementary Figure S5. S100A11 is not involved in the control of cell invasion in vivo. Expression of S100A11 was reduced by siRNA in PDAC cells, and the immunoblotting analysis presents the extent of protein depletion in this experiment. Invasion of control and S100A11-depleted cells was analysed in zebrafish embryo invasion assay. Results are mean +/- StDev of biological replicates (n=3). Cell invasion was analysed in 10 fish per condition in each experiment.

Supplementary Figure S6

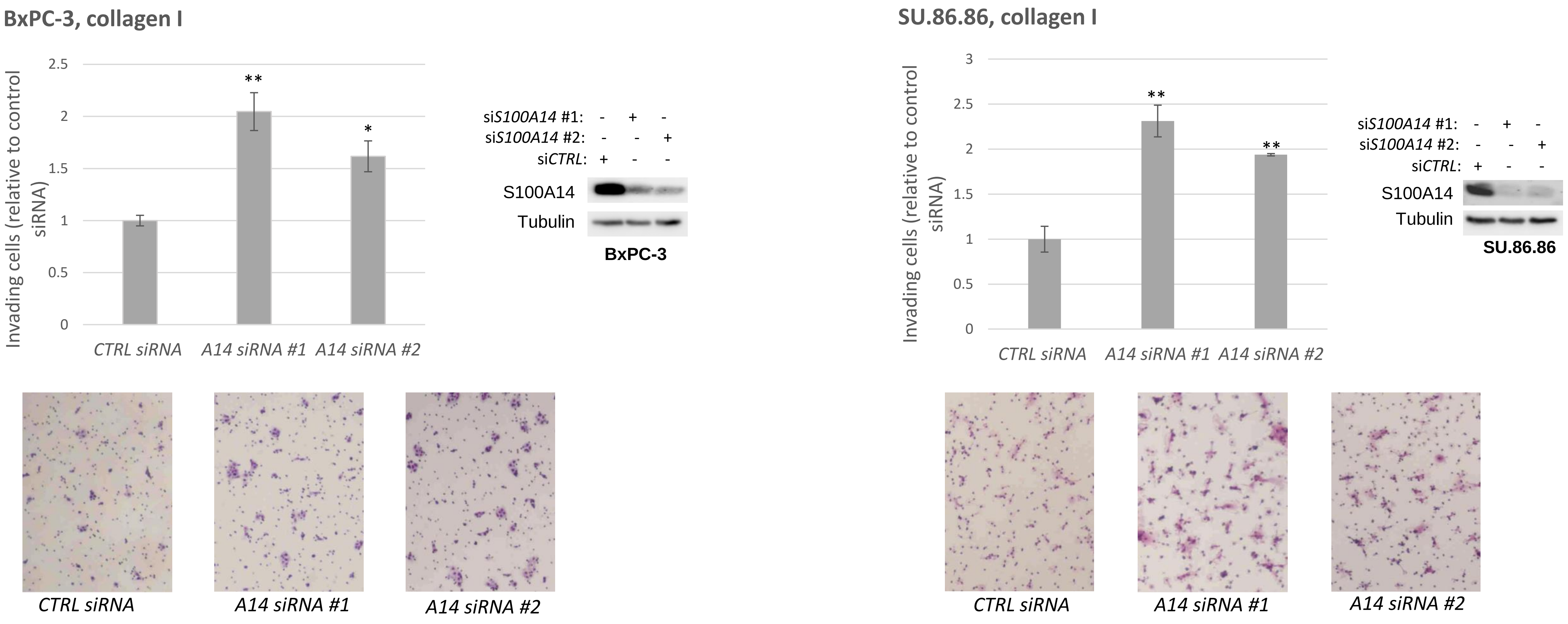

Supplementary Figure S6. S100A14 reduces invasive potential of epithelial PDAC cells *in vitro*. BxPC-3 and SU.86.86 cells were transfected with the control siRNA, or siRNA targeting S100A14 and seeded in Transwell inserts coated with collagen I. Cell invasion was analysed as described in Materials and Methods. Results are mean +/- StDev of triplicate experiments. *p<0.05; **p<0.01 (Student's t Test). The representative microscopy images of invaded cells are shown.

Supplementary Figure S7

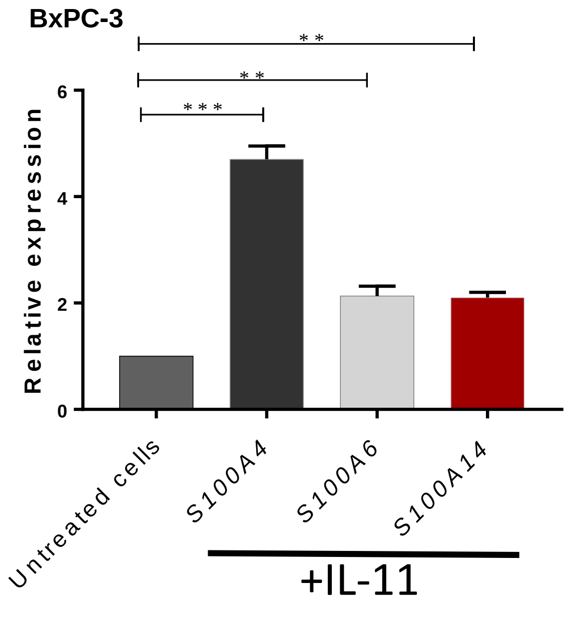

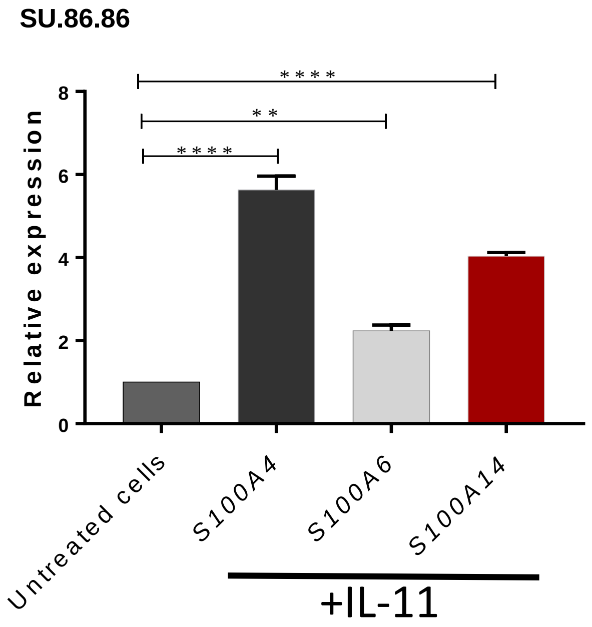

Supplementary Figure S7. Transcriptional up-regulation of *S100A4*, *S100A6* and *S100A14* genes in BxPC-3 and SU.86.86 cell lines in response to the treatment with IL-11. Cells were treated with IL-11 for 48 hours, or left untreated, and the transcription of indicated *S100* genes was analysed by qPCR. mRNA levels of *S100* genes in IL-11-treated cells was expressed as fold difference relative to untreated cells. Data represent means of three replicate measurements +/− StDev. **p<0.01; ***p<0.001; ****p<0.0001 (Student's t Test).

Supplementary Figure S8

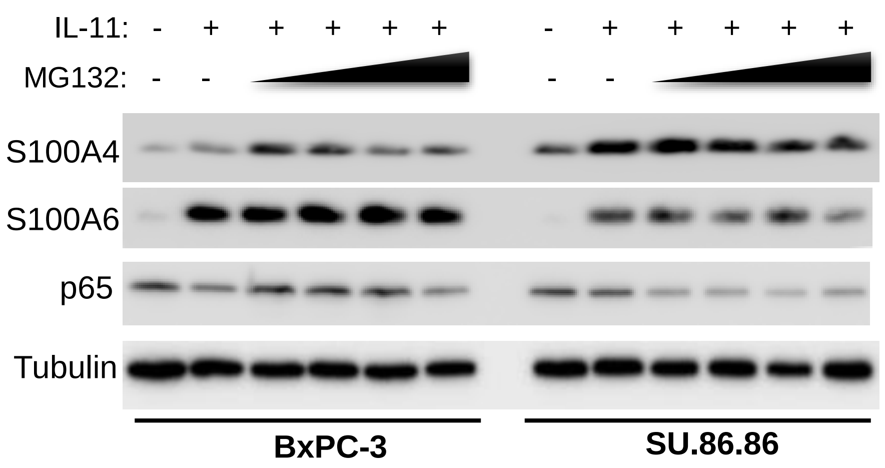

Supplementary Figure S8. NF-κB has no role in IL-11-induced activation of *S100* genes in epithelial PDAC cells. BxPC-3 and SU.86.86 cell lines were treated with IL-11 or left untreated. 16 hours before harvesting, cells grown in the presence of IL-11 were treated with the increasing concentrations of MG132 (6, 8, 10 and 12 μM), which acts as a non-specific inhibitor of NF-κB. This treatment did not affect the expression levels of S100A4 or S100A6 proteins.

Supplementary Table S1. siRNA oligonucleotides

siRNA	Supplier	Catalogue number/siRNA ID
Non-Targeting	Dharmacon	D-001810-01-05
S100A4 #1	Dharmacon	L-004792-00-0005
S100A4 #2	Ambion	ID s12319
S100A6 #1	Dharmacon	L-013463-00-0005
S100A6 #2	Ambion	ID s12418
S100A14 #1	Ambion	ID s32990
S100A14 #2	Ambion	ID s32989
S100A11	Sigma-Aldrich	4192015
STAT3	Dharmacon	L-003544-00-0005

Supplementary Table S2. Primer sequences used for qPCR

Gene name	5'-Primers	3'-Primers
ZEB1	GATGACCTGCCAACAGACCA	CTTTCACTGCTCCTCCCTGG
CDH1	ATGGCTGAAGGTGACAGAGC	TGCATTCCCGTTGGATGACA
CDH3	ACCAACCATCATCCCGACAC	GTTAGCCGCCTTCAGGTTCT
VIM	CTCTGGCACGTCTTGACCTT	GCCATCAACCTCTTCGTGGA
S100A2	CCAGCTTTGTGGGGGAGAAA	TGAGTGCCAGGAAAACAGCA
S100A4	CTAAAGGAGCTGCTGACCCG	TGTCCCTGTTGCTGTCCAAG
S100A6	GAAGGAGCTCACCATTGGCT	CACCTCCTGGTCCTTGTTCC
S100A7	ACCTCGCCGATGTCTTTGAG	CCATGGCTCTGCTTGTGGTA
S100A8	AAGGGGAATTTCCATGCCGT	AGGACACTCGGTCTCTAGCA
S100A9	GCTGGTGCGAAAAGATCTGC	TGTGTCCAGGTCCTCCATGA
S100A10	AAAAGACCCTCTGGCTGTGG	AATGGTGAGGCCCGCAATTA
S100A11	GGTGTCCTTGACCGCATGAT	AGGAAGGAGTCATGGCAAGC
S100A13	ATCTGCTCAAGGATGTGGGC	GCCAGCTCCCCAATCAATCT
S100A14	CGCAGAGGATGCTCAGGAAT	GTAGCTCAGAAGGGGTCAGC
S100P	GCTCAAGGTGCTGATGGAGA	CAGCCACGAACACGATGAAC
GAPDH	GTCAAGGCTGAGAACGGGAA	CAGCCACGAACACGATGAAC

Supplementary Table S3. Statistical analysis of S100A4 expression in CP (Group 1.00), PanIN (Group 2.00) and PDAC (Group 3.00) (Kruskal Wallis Test).

	Group	N	Mean Rank
S100A4	1.00	22	18.91
	2.00	18	24.28
	3.00	8	40.38
	Total	48	

Test Statistics[a,b]

	S100A4
Chi-Square	22.741
df	2
Asymp. Sig.	.000

a. Kruskal Wallis Test
b. Grouping Variable: group

Supplementary Table S4. Statistical analysis of S100A6 expression in CP (Group 1.00), PanIN (Group 2.00) and PDAC (Group 3.00) (Kruskal Wallis Test).

	Group	N	Mean Rank
S100A6	1.00	22	14.48
	2.00	18	30.69
	3.00	8	38.13
	Total	48	

Test Statistics[a,b]

	S100A6
Chi-Square	24.941
df	2
Asymp. Sig.	.000

a. Kruskal Wallis Test
b. Grouping Variable: group
